# Supplementary material for: Hypolipidemic Effect of Arthrospira (Spirulina) maxima Supplementation and a Systematic Physical Exercise Program in Overweight and Obese Men: A Double-Blind, Randomized, and Crossover Controlled Trial
Source: Mar Drugs. 2019 May 7;17(5):270. doi: 10.3390/md17050270 (PMC6562443; doi:10.3390/md17050270)

CONSORT
TRANSPARENT REPORTING of TRIALS

Supplementary File 2. CONSORT 2010 Flow Diagram of the progress through the phases of the trial.

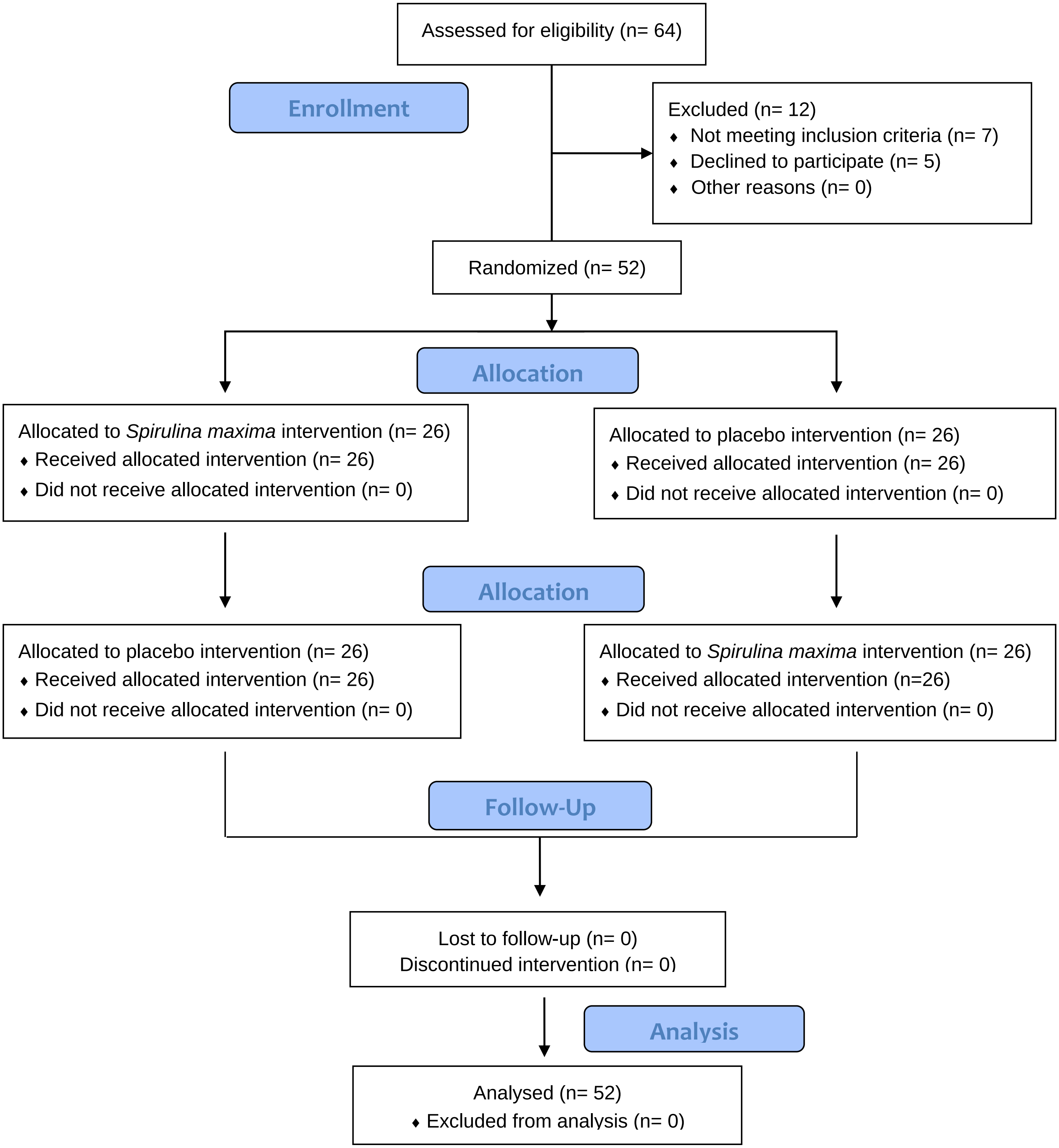

Supplement: Supplementary file 1 [file marinedrugs-17-00270-s001.zip › Supplementary Files/Supplementary File 2.pdf]
