# Supplementary material for: Hypolipidemic Effect of Arthrospira (Spirulina) maxima Supplementation and a Systematic Physical Exercise Program in Overweight and Obese Men: A Double-Blind, Randomized, and Crossover Controlled Trial
Source: Mar Drugs. 2019 May 7;17(5):270. doi: 10.3390/md17050270 (PMC6562443; doi:10.3390/md17050270)
Supplement: Supplementary file 1 [file marinedrugs-17-00270-s001.zip › Supplementary Files/Supplementary File 3.pdf]

Supplementary File 3. Statistical Analysis

Table 2

Effect of **C** on **TC** within the total of participants

Independent Samples Test

		Levene's Test for Equality of Variances		t-test for Equality of Means					95% Confidence Interval of the Difference	
		F	Sig.	t	df	Sig. (2-tailed)	Mean Difference	Std. Error Difference	Lower	Upper
TC	Equal variances assumed	.042	.839	.501	46	.619	4.50000	8.97772	-13.57122	22.57122
	Equal variances not assumed			.501	45.994	.619	4.50000	8.97772	-13.57128	22.57128

Effect of **SE** on **TC** within the total of participants

Independent Samples Test

		Levene's Test for Equality of Variances		t-test for Equality of Means					95% Confidence Interval of the Difference	
		F	Sig.	t	df	Sig. (2-tailed)	Mean Difference	Std. Error Difference	Lower	Upper
TC	Equal variances assumed	.129	.721	3.677	52	.001	33.74074	9.17529	15.32918	52.15230
	Equal variances not assumed			3.677	51.786	.001	33.74074	9.17529	15.32737	52.15411

Effect of **Ex** on **TC** within the total of participants

Independent Samples Test

		Levene's Test for Equality of Variances		t-test for Equality of Means					95% Confidence Interval of the Difference	
		F	Sig.	t	df	Sig. (2-tailed)	Mean Difference	Std. Error Difference	Lower	Upper
TC	Equal variances assumed	.296	.588	1.977	52	.053	19.81481	10.02430	-.30042	39.93005
	Equal variances not assumed			1.977	51.753	.053	19.81481	10.02430	-.30271	39.93233

Effect of **Sm** on **TC** within the total of participants

Independent Samples Test

		Levene's Test for Equality of Variances		t-test for Equality of Means					95% Confidence Interval of the Difference	
		F	Sig.	t	df	Sig. (2-tailed)	Mean Difference	Std. Error Difference	Lower	Upper
TC	Equal variances assumed	.238	.628	1.665	46	.103	18.20833	10.93908	-3.81090	40.22757
	Equal variances not assumed			1.665	45.950	.103	18.20833	10.93908	-3.81155	40.22821

Effect of **C** on **TG** within the total of participants

Independent Samples Test

		Levene's Test for Equality of Variances		t-test for Equality of Means					95% Confidence Interval of the Difference	
		F	Sig.	t	df	Sig. (2-tailed)	Mean Difference	Std. Error Difference	Lower	Upper
TG	Equal variances assumed	.070	.793	.653	44	.517	6.04348	9.25740	-12.61358	24.70054
	Equal variances not assumed			.653	43.973	.517	6.04348	9.25740	-12.61391	24.70086

Effect of **SE** on **TG** within the total of participants

Independent Samples Test

		Levene's Test for Equality of Variances		t-test for Equality of Means						
									95% Confidence Interval of the Difference	
		F	Sig.	t	df	Sig. (2-tailed)	Mean Difference	Std. Error Difference	Lower	Upper
TG	Equal variances assumed	.362	.550	1.853	46	.070	22.50000	12.14025	-1.93706	46.93706
	Equal variances not assumed			1.853	43.368	.071	22.50000	12.14025	-1.97714	46.97714

Effect of **Ex** on **TG** within the total of participants

Independent Samples Test

		Levene's Test for Equality of Variances		t-test for Equality of Means						
									95% Confidence Interval of the Difference	
		F	Sig.	t	df	Sig. (2-tailed)	Mean Difference	Std. Error Difference	Lower	Upper
TG	Equal variances assumed	.033	.857	1.263	50	.212	15.11538	11.96518	-8.91738	39.14815
	Equal variances not assumed			1.263	49.946	.212	15.11538	11.96518	-8.91803	39.14880

Effect of **Sm** on **TG** within the total of participants

Independent Samples Test

		Levene's Test for Equality of Variances		t-test for Equality of Means						
									95% Confidence Interval of the Difference	
		F	Sig.	t	df	Sig. (2-tailed)	Mean Difference	Std. Error Difference	Lower	Upper
TG	Equal variances assumed	.160	.691	1.374	46	.176	14.12500	10.27909	-6.56573	34.81573
	Equal variances not assumed			1.374	45.736	.176	14.12500	10.27909	-6.56895	34.81895

Effect of **C** on **LDL-C** within the total of participants

Independent Samples Test

		Levene's Test for Equality of Variances		t-test for Equality of Means						
									95% Confidence Interval of the Difference	
		F	Sig.	t	df	Sig. (2-tailed)	Mean Difference	Std. Error Difference	Lower	Upper
LDL	Equal variances assumed	.199	.658	.416	42	.680	3.90000	9.38077	-15.03117	22.83117
	Equal variances not assumed			.416	41.906	.680	3.90000	9.38077	-15.03243	22.83243

Effect of **SE** on **LDL-C** within the total of participants

Independent Samples Test

		Levene's Test for Equality of Variances		t-test for Equality of Means						
									95% Confidence Interval of the Difference	
		F	Sig.	t	df	Sig. (2-tailed)	Mean Difference	Std. Error Difference	Lower	Upper
LDL	Equal variances assumed	.031	.861	3.461	46	.001	34.62500	10.00352	14.48896	54.76104
	Equal variances not assumed			3.461	45.876	.001	34.62500	10.00352	14.48748	54.76252

Effect of **Ex** on **LDL-C** within the total of participants

Independent Samples Test

		Levene's Test for Equality of Variances		t-test for Equality of Means						
									95% Confidence Interval of the Difference	
		F	Sig.	t	df	Sig. (2-tailed)	Mean Difference	Std. Error Difference	Lower	Upper
LDL	Equal variances assumed	.291	.592	2.049	46	.046	21.96667	10.72233	.38373	43.54960
	Equal variances not assumed			2.049	45.860	.046	21.96667	10.72233	.38195	43.55138

Effect of **Sm** on **LDL-C** within the total of participants

Independent Samples Test

		Levene's Test for Equality of Variances		t-test for Equality of Means						
									95% Confidence Interval of the Difference	
		F	Sig.	t	df	Sig. (2-tailed)	Mean Difference	Std. Error Difference	Lower	Upper
LDL	Equal variances assumed	.004	.947	1.911	46	.062	20.84167	10.90583	-1.11063	42.79396
	Equal variances not assumed			1.911	45.941	.062	20.84167	10.90583	-1.11139	42.79472

Effect of **C** on **HDL-C** within the total of participants

Independent Samples Test

		Levene's Test for Equality of Variances		t-test for Equality of Means						
									95% Confidence Interval of the Difference	
		F	Sig.	t	df	Sig. (2-tailed)	Mean Difference	Std. Error Difference	Lower	Upper
HDL	Equal variances assumed	1.768	.190	-.450	44	.655	-1.34783	2.99475	-7.38336	4.68770
	Equal variances not assumed			-.450	40.175	.655	-1.34783	2.99475	-7.39963	4.70398

Effect of **SE** on **HDL-C** within the total of participants

Independent Samples Test

		Levene's Test for Equality of Variances		t-test for Equality of Means						
									95% Confidence Interval of the Difference	
		F	Sig.	t	df	Sig. (2-tailed)	Mean Difference	Std. Error Difference	Lower	Upper
HDL	Equal variances assumed	.058	.811	-2.898	52	.005	-7.33333	2.53014	-12.41043	-2.25624
	Equal variances not assumed			-2.898	50.962	.006	-7.33333	2.53014	-12.41289	-2.25378

Effect of **Ex** on **HDL-C** within the total of participants

Independent Samples Test

		Levene's Test for Equality of Variances		t-test for Equality of Means						
									95% Confidence Interval of the Difference	
		F	Sig.	t	df	Sig. (2-tailed)	Mean Difference	Std. Error Difference	Lower	Upper
HDL	Equal variances assumed	.009	.926	-1.930	48	.059	-4.72000	2.44524	-9.63649	.19649
	Equal variances not assumed			-1.930	47.974	.059	-4.72000	2.44524	-9.63655	.19655

Effect of **Sm** on **HDL-C** within the total of participants

Independent Samples Test

		Levene's Test for Equality of Variances		t-test for Equality of Means						
									95% Confidence Interval of the Difference	
		F	Sig.	t	df	Sig. (2-tailed)	Mean Difference	Std. Error Difference	Lower	Upper
HDL	Equal variances assumed	.073	.788	-1.849	46	.071	-5.45833	2.95190	-11.40019	.48353
	Equal variances not assumed			-1.849	45.392	.071	-5.45833	2.95190	-11.40234	.48567

Effect of **C** on **TC** within dyslipidemic participants

Independent Samples Test

		Levene's Test for Equality of Variances		t-test for Equality of Means						
									95% Confidence Interval of the Difference	
		F	Sig.	t	df	Sig. (2-tailed)	Mean Difference	Std. Error Difference	Lower	Upper
CT_Dyslip	Equal variances assumed	.183	.674	.841	18	.412	6.40000	7.61212	-9.59248	22.39248
	Equal variances not assumed			.841	17.507	.412	6.40000	7.61212	-9.62480	22.42480

Effect of **SE** on **TC** within dyslipidemic participants

Independent Samples Test

		Levene's Test for Equality of Variances		t-test for Equality of Means						
									95% Confidence Interval of the Difference	
		F	Sig.	t	df	Sig. (2-tailed)	Mean Difference	Std. Error Difference	Lower	Upper
CT_Dyslip	Equal variances assumed	1.130	.298	4.373	24	.000	36.30769	8.30348	19.17016	53.44523
	Equal variances not assumed			4.373	23.833	.000	36.30769	8.30348	19.16378	53.45160

Effect of **Ex** on **TC** within dyslipidemic participants

Independent Samples Test

		Levene's Test for Equality of Variances		t-test for Equality of Means						
									95% Confidence Interval of the Difference	
		F	Sig.	t	df	Sig. (2-tailed)	Mean Difference	Std. Error Difference	Lower	Upper
CT_Dyslip	Equal variances assumed	.238	.630	2.411	22	.025	24.91667	10.33526	3.48265	46.35068
	Equal variances not assumed			2.411	21.303	.025	24.91667	10.33526	3.44193	46.39140

Effect of **Sm** on **TC** within dyslipidemic participants

Independent Samples Test

		Levene's Test for Equality of Variances		t-test for Equality of Means						
									95% Confidence Interval of the Difference	
		F	Sig.	t	df	Sig. (2-tailed)	Mean Difference	Std. Error Difference	Lower	Upper
CT_Dyslip	Equal variances assumed	.691	.415	2.341	22	.029	21.16667	9.04248	2.41371	39.91962
	Equal variances not assumed			2.341	21.860	.029	21.16667	9.04248	2.40676	39.92658

Effect of **C** on **TG** within dyslipidemic participants

Independent Samples Test

		Levene's Test for Equality of Variances		t-test for Equality of Means						
									95% Confidence Interval of the Difference	
		F	Sig.	t	df	Sig. (2-tailed)	Mean Difference	Std. Error Difference	Lower	Upper
TG_Dyslip	Equal variances assumed	6.799	.019	1.520	16	.148	6.77778	4.45797	-2.67269	16.22824
	Equal variances not assumed			1.520	11.312	.156	6.77778	4.45797	-3.00119	16.55675

Effect of **SE** on **TG** within dyslipidemic participants

Independent Samples Test

		Levene's Test for Equality of Variances		t-test for Equality of Means						
									95% Confidence Interval of the Difference	
		F	Sig.	t	df	Sig. (2-tailed)	Mean Difference	Std. Error Difference	Lower	Upper
TG_Dyslip	Equal variances assumed	.129	.722	2.122	26	.043	28.00000	13.19317	.88106	55.11894
	Equal variances not assumed			2.122	23.976	.044	28.00000	13.19317	.76920	55.23080

Effect of **Ex** on **TG** within dyslipidemic participants

Independent Samples Test

		Levene's Test for Equality of Variances		t-test for Equality of Means						
									95% Confidence Interval of the Difference	
		F	Sig.	t	df	Sig. (2-tailed)	Mean Difference	Std. Error Difference	Lower	Upper
TG_Dyslip	Equal variances assumed	.224	.641	1.739	22	.096	16.25000	9.34695	-3.13439	35.63439
	Equal variances not assumed			1.739	21.397	.096	16.25000	9.34695	-3.16611	35.66611

Effect of **Sm** on **TG** within dyslipidemic participants

Independent Samples Test

		Levene's Test for Equality of Variances		t-test for Equality of Means						
									95% Confidence Interval of the Difference	
		F	Sig.	t	df	Sig. (2-tailed)	Mean Difference	Std. Error Difference	Lower	Upper
TG_Dyslip	Equal variances assumed	1.199	.284	3.142	24	.004	19.30769	6.14527	6.62449	31.99090
	Equal variances not assumed			3.142	18.655	.005	19.30769	6.14527	6.42939	32.18600

Effect of **C** on **LDL-C** within dyslipidemic participants

Independent Samples Test

		Levene's Test for Equality of Variances		t-test for Equality of Means						
									95% Confidence Interval of the Difference	
		F	Sig.	t	df	Sig. (2-tailed)	Mean Difference	Std. Error Difference	Lower	Upper
LDL_Dyslip	Equal variances assumed	.417	.523	.458	34	.650	4.30000	9.38776	-14.77822	23.37822
	Equal variances not assumed			.458	33.869	.650	4.30000	9.38776	-14.78093	23.38093

Effect of **SE** on **LDL-C** within dyslipidemic participants

Independent Samples Test

		Levene's Test for Equality of Variances		t-test for Equality of Means						
									95% Confidence Interval of the Difference	
		F	Sig.	t	df	Sig. (2-tailed)	Mean Difference	Std. Error Difference	Lower	Upper
LDL_Dyslip	Equal variances assumed	.860	.360	3.767	34	.001	40.02222	10.62349	18.43270	61.61174
	Equal variances not assumed			3.767	33.203	.001	40.02222	10.62349	18.41360	61.63084

Effect of **Ex** on **LDL-C** within dyslipidemic participants

Independent Samples Test

		Levene's Test for Equality of Variances		t-test for Equality of Means						
									95% Confidence Interval of the Difference	
		F	Sig.	t	df	Sig. (2-tailed)	Mean Difference	Std. Error Difference	Lower	Upper
LDL_Dyslip	Equal variances assumed	.002	.964	2.261	38	.030	23.64000	10.45350	2.47800	44.80200
	Equal variances not assumed			2.261	37.998	.030	23.64000	10.45350	2.47796	44.80204

Effect of **Sm** on **LDL-C** within dyslipidemic participants

Independent Samples Test

		Levene's Test for Equality of Variances		t-test for Equality of Means						
									95% Confidence Interval of the Difference	
		F	Sig.	t	df	Sig. (2-tailed)	Mean Difference	Std. Error Difference	Lower	Upper
LDL_Dyslip	Equal variances assumed	.051	.823	1.936	38	.060	19.78000	10.21901	-.90731	40.46731
	Equal variances not assumed			1.936	37.962	.060	19.78000	10.21901	-.90798	40.46798

Effect of **C** on **HDL-C** within dyslipidemic participants

Independent Samples Test

		Levene's Test for Equality of Variances		t-test for Equality of Means						
									95% Confidence Interval of the Difference	
		F	Sig.	t	df	Sig. (2-tailed)	Mean Difference	Std. Error Difference	Lower	Upper
HDL_Dyslip	Equal variances assumed	7.309	.012	-1.415	26	.169	-3.35714	2.37241	-8.23370	1.51942
	Equal variances not assumed			-1.415	21.295	.172	-3.35714	2.37241	-8.28669	1.57240

Effect of **SE** on **HDL-C** within dyslipidemic participants

Independent Samples Test

		Levene's Test for Equality of Variances		t-test for Equality of Means						
									95% Confidence Interval of the Difference	
		F	Sig.	t	df	Sig. (2-tailed)	Mean Difference	Std. Error Difference	Lower	Upper
HDL_Dyslip	Equal variances assumed	1.321	.258	-3.652	38	.001	-9.05000	2.47830	-14.06706	-4.03294
	Equal variances not assumed			-3.652	30.721	.001	-9.05000	2.47830	-14.10639	-3.99361

Effect of **Ex** on **HDL-C** within dyslipidemic participants

Independent Samples Test

		Levene's Test for Equality of Variances		t-test for Equality of Means					95% Confidence Interval of the Difference	
		F	Sig.	t	df	Sig. (2-tailed)	Mean Difference	Std. Error Difference	Lower	Upper
HDL_Dyslip	Equal variances assumed	.015	.904	-2.445	38	.019	-4.95000	2.02468	-9.04876	-.85124
	Equal variances not assumed			-2.445	37.999	.019	-4.95000	2.02468	-9.04876	-.85124

Effect of **Sm** on **HDL-C** within dyslipidemic participants

Independent Samples Test

		Levene's Test for Equality of Variances		t-test for Equality of Means					95% Confidence Interval of the Difference	
		F	Sig.	t	df	Sig. (2-tailed)	Mean Difference	Std. Error Difference	Lower	Upper
HDL_Dyslip	Equal variances assumed	2.542	.122	-2.158	28	.040	-6.66667	3.08925	-12.99472	-.33862
	Equal variances not assumed			-2.158	21.849	.042	-6.66667	3.08925	-13.07595	-.25738

Figures 1-3
Overweight

Hypothesis Test Summary

	Null Hypothesis	Test	Sig.	Decision
1	The distribution of Dif_TC is the same across categories of Overweight.	Independent-Samples Kruskal-Wallis Test	.000	Reject the null hypothesis.
2	The distribution of Dif_TG is the same across categories of Overweight.	Independent-Samples Kruskal-Wallis Test	.002	Reject the null hypothesis.
3	The distribution of Dif_LDL is the same across categories of Overweight.	Independent-Samples Kruskal-Wallis Test	.000	Reject the null hypothesis.
4	The distribution of Dif_HDL is the same across categories of Overweight.	Independent-Samples Kruskal-Wallis Test	.016	Reject the null hypothesis.
5	The distribution of Dif_BMI is the same across categories of Overweight.	Independent-Samples Kruskal-Wallis Test	.133	Retain the null hypothesis.

Asymptotic significances are displayed. The significance level is .05.

Obese

Hypothesis Test Summary

	Null Hypothesis	Test	Sig.	Decision
1	The distribution of Dif_TC is the same across categories of Obesity.	Independent-Samples Kruskal-Wallis Test	.000	Reject the null hypothesis.
2	The distribution of Dif_TG is the same across categories of Obesity.	Independent-Samples Kruskal-Wallis Test	.328	Retain the null hypothesis.
3	The distribution of Dif_LDL is the same across categories of Obesity.	Independent-Samples Kruskal-Wallis Test	.000	Reject the null hypothesis.
4	The distribution of Dif_HDL is the same across categories of Obesity.	Independent-Samples Kruskal-Wallis Test	.236	Retain the null hypothesis.
5	The distribution of Dif_BMI is the same across categories of Obesity.	Independent-Samples Kruskal-Wallis Test	.000	Reject the null hypothesis.

Asymptotic significances are displayed. The significance level is .05.

Dyslipidemic

Hypothesis Test Summary

	Null Hypothesis	Test	Sig.	Decision
1	The distribution of Dif_CT_Dyslip is the same across categories of Group_TOTAL.	Independent-Samples Kruskal-Wallis Test	.000	Reject the null hypothesis.

	Null Hypothesis	Test	Sig.	Decision
1	The distribution of Dif_TG_Dyslip is the same across categories of Group_TOTAL.	Independent-Samples Kruskal-Wallis Test	.003	Reject the null hypothesis.

	Null Hypothesis	Test	Sig.	Decision
1	The distribution of Dif_LDL_Dyslip is the same across categories of Group_TOTAL.	Independent-Samples Kruskal-Wallis Test	.000	Reject the null hypothesis.

	Null Hypothesis	Test	Sig.	Decision
1	The distribution of Dif_HDL_Dyslip is the same across categories of Group_TOTAL.	Independent-Samples Kruskal-Wallis Test	.037	Reject the null hypothesis.

	Null Hypothesis	Test	Sig.	Decision
1	The distribution of Dif_BMI_Dyslip is the same across categories of Group_TOTAL.	Independent-Samples Kruskal-Wallis Test	.000	Reject the null hypothesis.

Asymptotic significances are displayed. The significance level is .05.

TC. Overweight

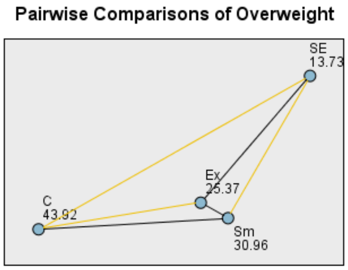

Each node shows the sample average rank of Overweight.

Sample1-Sample2	Test Statistic	Std. Error	Std. Test Statistic	Sig.	Adj.Sig.
SE-Ex	-11.633	5.740	-2.027	.043	.256
SE-Sm	-17.225	6.088	-2.829	.005	.028
SE-C	-30.183	6.088	-4.958	.000	.000
Ex-Sm	-5.592	6.088	-.918	.358	1.000
Ex-C	-18.550	6.088	-3.047	.002	.014
Sm-C	-12.958	6.417	-2.019	.043	.261

Each row tests the null hypothesis that the Sample 1 and Sample 2 distributions are the same.
Asymptotic significances (2-sided tests) are displayed. The significance level is .05.

TC. Obese

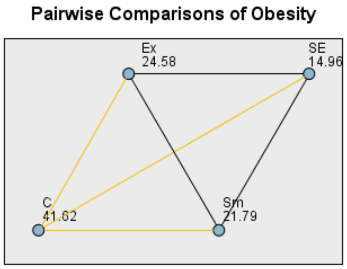

Each node shows the sample average rank of Obesity.

Sample1-Sample2	Test Statistic	Std. Error	Std. Test Statistic	Sig.	Adj.Sig.
SE-Sm	-6.830	5.833	-1.171	.242	1.000
SE-Ex	-9.615	5.715	-1.682	.092	.555
SE-C	-26.663	5.833	-4.571	.000	.000
Sm-Ex	2.785	5.833	.478	.633	1.000
Sm-C	-19.833	5.948	-3.334	.001	.005
Ex-C	-17.048	5.833	-2.923	.003	.021

Each row tests the null hypothesis that the Sample 1 and Sample 2 distributions are the same.
Asymptotic significances (2-sided tests) are displayed. The significance level is .05.

TC. Dyslipidemic

Pairwise Comparisons of Group_TOTAL

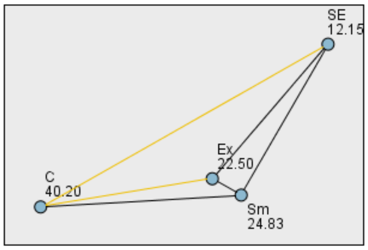

Each node shows the sample average rank of Group_TOTAL.

Sample1-Sample2	Test Statistic	Std. Error	Std. Test Statistic	Sig.	Adj.Sig.
SE-Ex	-10.346	5.486	-1.886	.059	.356
SE-Sm	-12.679	5.486	-2.311	.021	.125
SE-C	-28.046	5.764	-4.866	.000	.000
Ex-Sm	-2.333	5.594	-.417	.677	1.000
Ex-C	-17.700	5.867	-3.017	.003	.015
Sm-C	-15.367	5.867	-2.619	.009	.053

Each row tests the null hypothesis that the Sample 1 and Sample 2 distributions are the same.
Asymptotic significances (2-sided tests) are displayed. The significance level is .05.

TG. Overweight

Pairwise Comparisons of Overweight

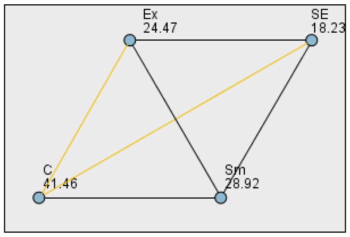

Each node shows the sample average rank of Overweight.

Sample1-Sample2	Test Statistic	Std. Error	Std. Test Statistic	Sig.	Adj.Sig.
SE-Ex	-6.233	5.736	-1.087	.277	1.000
SE-Sm	-10.683	6.084	-1.756	.079	.475
SE-C	-23.225	6.084	-3.817	.000	.001
Ex-Sm	-4.450	6.084	-.731	.465	1.000
Ex-C	-16.992	6.084	-2.793	.005	.031
Sm-C	-12.542	6.413	-1.956	.051	.303

Each row tests the null hypothesis that the Sample 1 and Sample 2 distributions are the same.
Asymptotic significances (2-sided tests) are displayed. The significance level is .05.

TG. Obese

ANOVA

Dif_TG

	Sum of Squares	df	Mean Square	F	Sig.
Between Groups	1525.592	3	508.531	.776	.513
Within Groups	30133.628	46	655.079		
Total	31659.220	49			

Multiple Comparisons

Dependent Variable: Dif_TG

Tukey HSD

(I) Obesity	(J) Obesity	Mean Difference (I-J)	Std. Error	Sig.	95% Confidence Interval	
					Lower Bound	Upper Bound
SE	Ex	8.53846	10.03899	.830	-18.2204	35.2974
	Sm	14.44231	10.24600	.500	-12.8684	41.7530
	C	2.77564	10.24600	.993	-24.5350	30.0863
Ex	SE	-8.53846	10.03899	.830	-35.2974	18.2204
	Sm	5.90385	10.24600	.939	-21.4068	33.2145
	C	-5.76282	10.24600	.943	-33.0735	21.5479
Sm	SE	-14.44231	10.24600	.500	-41.7530	12.8684
	Ex	-5.90385	10.24600	.939	-33.2145	21.4068
	C	-11.66667	10.44891	.681	-39.5182	16.1849
C	SE	-2.77564	10.24600	.993	-30.0863	24.5350
	Ex	5.76282	10.24600	.943	-21.5479	33.0735
	Sm	11.66667	10.44891	.681	-16.1849	39.5182

TG. Dyslipidemic

Pairwise Comparisons of Group_TOTAL

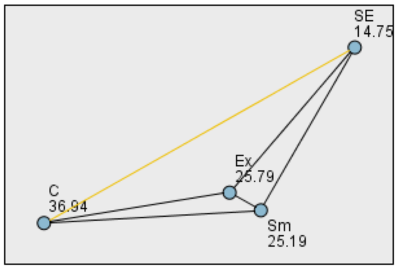

Each node shows the sample average rank of Group_TOTAL.

Sample1-Sample2	Test Statistic	Std. Error	Std. Test Statistic	Sig.	Adj.Sig.
SE-Sm	-10.442	5.388	-1.938	.053	.316
SE-Ex	-11.042	5.503	-2.006	.045	.269
SE-C	-22.194	5.977	-3.713	.000	.001
Sm-Ex	.599	5.600	.107	.915	1.000
Sm-C	-11.752	6.066	-1.937	.053	.316
Ex-C	-11.153	6.169	-1.808	.071	.424

Each row tests the null hypothesis that the Sample 1 and Sample 2 distributions are the same.
Asymptotic significances (2-sided tests) are displayed. The significance level is .05.

LDL-C. Overweight

Pairwise Comparisons of Overweight

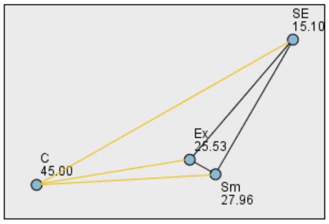

Each node shows the sample average rank of Overweight.

Sample1-Sample2	Test Statistic	Std. Error	Std. Test Statistic	Sig.	Adj.Sig.
SE-Ex	-10.433	5.743	-1.817	.069	.416
SE-Sm	-12.858	6.092	-2.111	.035	.209
SE-C	-29.900	6.092	-4.908	.000	.000
Ex-Sm	-2.425	6.092	-.398	.691	1.000
Ex-C	-19.467	6.092	-3.196	.001	.008
Sm-C	-17.042	6.421	-2.654	.008	.048

Each row tests the null hypothesis that the Sample 1 and Sample 2 distributions are the same.
Asymptotic significances (2-sided tests) are displayed. The significance level is .05.

LDL-C. Obese

Pairwise Comparisons of Obesity

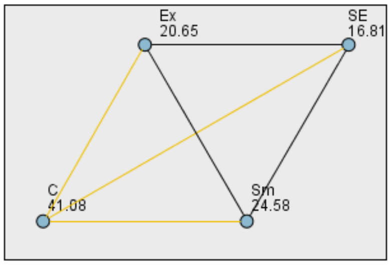

Each node shows the sample average rank of Obesity.

Sample1-Sample2	Test Statistic	Std. Error	Std. Test Statistic	Sig.	Adj.Sig.
SE-Ex	-3.846	5.718	-.673	.501	1.000
SE-Sm	-7.776	5.835	-1.332	.183	1.000
SE-C	-24.276	5.835	-4.160	.000	.000
Ex-Sm	-3.929	5.835	-.673	.501	1.000
Ex-C	-20.429	5.835	-3.501	.000	.003
Sm-C	-16.500	5.951	-2.773	.006	.033

Each row tests the null hypothesis that the Sample 1 and Sample 2 distributions are the same.
Asymptotic significances (2-sided tests) are displayed. The significance level is .05.

LDL-C. Dyslipidemic

Pairwise Comparisons of Group_TOTAL

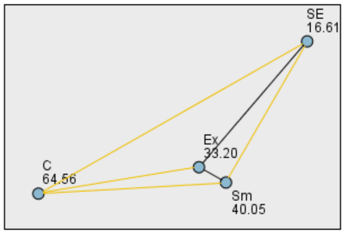

Each node shows the sample average rank of Group_TOTAL.

Sample1-Sample2	Test Statistic	Std. Error	Std. Test Statistic	Sig.	Adj.Sig.
SE-Ex	-16.589	7.174	-2.312	.021	.125
SE-Sm	-23.439	7.174	-3.267	.001	.007
SE-C	-47.944	7.361	-6.514	.000	.000
Ex-Sm	-6.850	6.983	-.981	.327	1.000
Ex-C	-31.356	7.174	-4.370	.000	.000
Sm-C	-24.506	7.174	-3.416	.001	.004

Each row tests the null hypothesis that the Sample 1 and Sample 2 distributions are the same.
Asymptotic significances (2-sided tests) are displayed. The significance level is .05.

HDL-C. Overweight

Pairwise Comparisons of Overweight

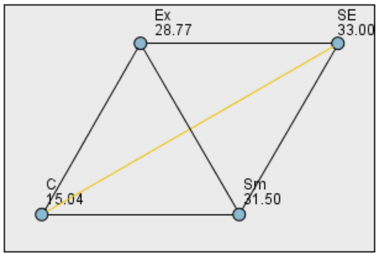

Each node shows the sample average rank of Overweight.

Sample1-Sample2	Test Statistic	Std. Error	Std. Test Statistic	Sig.	Adj.Sig.
C-Ex	13.725	6.066	2.262	.024	.142
C-Sm	16.458	6.395	2.574	.010	.060
C-SE	17.958	6.066	2.960	.003	.018
Ex-Sm	-2.733	6.066	-.451	.652	1.000
Ex-SE	4.233	5.720	.740	.459	1.000
Sm-SE	1.500	6.066	.247	.805	1.000

Each row tests the null hypothesis that the Sample 1 and Sample 2 distributions are the same.
Asymptotic significances (2-sided tests) are displayed. The significance level is .05.

HDL-C. Obese

ANOVA

Dif_HDL

	Sum of Squares	df	Mean Square	F	Sig.
Between Groups	575.143	3	191.714	1.346	.271
Within Groups	6552.077	46	142.436		
Total	7127.220	49			

Multiple Comparisons

Dependent Variable: Dif_HDL

Tukey HSD

(I) Obesity	(J) Obesity	Mean Difference (I-J)	Std. Error	Sig.	95% Confidence Interval Lower Bound	Upper Bound
SE	Ex	-2.38462	4.68116	.956	-14.8622	10.0930
	Sm	5.11538	4.77769	.709	-7.6195	17.8503
	C	5.61538	4.77769	.645	-7.1195	18.3503
Ex	SE	2.38462	4.68116	.956	-10.0930	14.8622
	Sm	7.50000	4.77769	.406	-5.2349	20.2349
	C	8.00000	4.77769	.349	-4.7349	20.7349
Sm	SE	-5.11538	4.77769	.709	-17.8503	7.6195
	Ex	-7.50000	4.77769	.406	-20.2349	5.2349
	C	.50000	4.87231	1.000	-12.4871	13.4871
C	SE	-5.61538	4.77769	.645	-18.3503	7.1195
	Ex	-8.00000	4.77769	.349	-20.7349	4.7349
	Sm	-.50000	4.87231	1.000	-13.4871	12.4871

HDL-C. Dyslipidemic

Pairwise Comparisons of Group_TOTAL

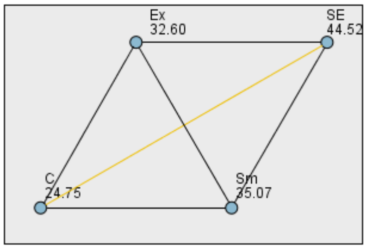

Each node shows the sample average rank of Group_TOTAL.

Sample1-Sample2	Test Statistic	Std. Error	Std. Test Statistic	Sig.	Adj.Sig.
C-Ex	7.850	6.972	1.126	.260	1.000
C-Sm	10.317	7.436	1.387	.165	.992
C-SE	19.775	6.972	2.836	.005	.027
Ex-Sm	-2.467	6.834	-.361	.718	1.000
Ex-SE	11.925	6.327	1.885	.059	.357
Sm-SE	9.458	6.834	1.384	.166	.998

Each row tests the null hypothesis that the Sample 1 and Sample 2 distributions are the same.
Asymptotic significances (2-sided tests) are displayed. The significance level is .05.

BMI. Overweight

ANOVA

Dif_BMI

	Sum of Squares	df	Mean Square	F	Sig.
Between Groups	1.918	3	.639	2.706	.055
Within Groups	11.812	50	.236		
Total	13.730	53			

Multiple Comparisons

Dependent Variable: Dif_BMI
Tukey HSD

(I) Overweight	(J) Overweight	Mean Difference (I-J)	Std. Error	Sig.	95% Confidence Interval	
					Lower Bound	Upper Bound
C	SE	-.47674*	.17748	.047	-.9484	-.0051
	Ex	-.18188	.18825	.769	-.6822	.3184
	Sm	-.36340	.18825	.229	-.8637	.1369
SE	C	.47674*	.17748	.047	.0051	.9484
	Ex	.29486	.18825	.407	-.2054	.7951
	Sm	.11333	.18825	.931	-.3869	.6136
Ex	C	.18188	.18825	.769	-.3184	.6822
	SE	-.29486	.18825	.407	-.7951	.2054
	Sm	-.18153	.19843	.797	-.7089	.3458
Sm	C	.36340	.18825	.229	-.1369	.8637
	SE	-.11333	.18825	.931	-.6136	.3869
	Ex	.18153	.19843	.797	-.3458	.7089

*. The mean difference is significant at the 0.05 level.

BMI. Obese

Pairwise Comparisons of Obesity

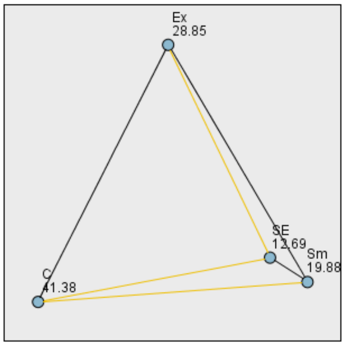

Each node shows the sample average rank of Obesity.

Sample1-Sample2	Test Statistic	Std. Error	Std. Test Statistic	Sig.	Adj.Sig.
SE-Sm	-7.183	5.823	-1.233	.217	1.000
SE-Ex	-16.154	5.706	-2.831	.005	.028
SE-C	-28.683	5.823	-4.926	.000	.000
Sm-Ex	8.971	5.823	1.541	.123	.741
Sm-C	-21.500	5.939	-3.620	.000	.002
Ex-C	-12.529	5.823	-2.152	.031	.189

Each row tests the null hypothesis that the Sample 1 and Sample 2 distributions are the same.
Asymptotic significances (2-sided tests) are displayed. The significance level is .05.

BMI. Dyslipidemic

Pairwise Comparisons of Group_TOTAL

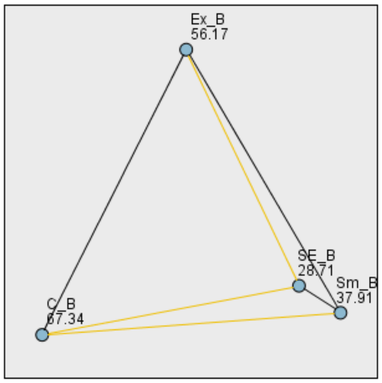

Each node shows the sample average rank of Group_TOTAL.

Sample1-Sample2	Test Statistic	Std. Error	Std. Test Statistic	Sig.	Adj.Sig.
SE_B-Sm_B	-9.201	8.035	-1.145	.252	1.000
SE_B-Ex_B	-27.465	7.706	-3.564	.000	.002
SE_B-C_B	-38.633	8.035	-4.808	.000	.000
Sm_B-Ex_B	18.264	7.886	2.316	.021	.123
Sm_B-C_B	-29.432	8.208	-3.586	.000	.002
Ex_B-C_B	-11.168	7.886	-1.416	.157	.940

Each row tests the null hypothesis that the Sample 1 and Sample 2 distributions are the same.
Asymptotic significances (2-sided tests) are displayed. The significance level is .05.

Table 1. Statistical analysis of baseline characteristics of subjects at the starting point of day 0 and after the wash-out period (day 56)

Group Statistics

	Session	N	Mean	Std. Deviation	Std. Error Mean
BMI	First session	52	30.2994	4.04770	.56131
	Third session	52	30.1011	4.03404	.55942
TC	First session	51	196.0196	34.65054	4.85205
	Third session	51	196.4118	36.66179	5.13368
TG	First session	51	141.4118	43.08697	6.03338
	Third session	51	145.4118	37.55166	5.25828
LDL	First session	51	135.5725	33.78241	4.73048
	Third session	51	132.8784	35.65912	4.99328
HDL	First session	51	33.6275	10.00592	1.40111
	Third session	51	34.2353	8.95229	1.25357

Independent Samples Test

		Levene's Test for Equality of Variances		t-test for Equality of Means					95% Confidence Interval of the Difference	
		F	Sig.	t	df	Sig. (2-tailed)	Mean Difference	Std. Error Difference	Lower	Upper
BMI	Equal variances assumed	.000	.990	.250	102	.803	.19830	.79248	-1.37358	1.77018
	Equal variances not assumed			.250	101.999	.803	.19830	.79248	-1.37358	1.77018
TC	Equal variances assumed	.613	.435	-.056	100	.956	-.39216	7.06378	-14.40650	13.62218
	Equal variances not assumed			-.056	99.683	.956	-.39216	7.06378	-14.40704	13.62273
TG	Equal variances assumed	.736	.393	-.500	100	.618	-4.00000	8.00320	-19.87813	11.87813
	Equal variances not assumed			-.500	98.167	.618	-4.00000	8.00320	-19.88176	11.88176
LDL	Equal variances assumed	.593	.443	.392	100	.696	2.69412	6.87825	-10.95213	16.34036
	Equal variances not assumed			.392	99.709	.696	2.69412	6.87825	-10.95262	16.34085
HDL	Equal variances assumed	.402	.527	-.323	100	.747	-.60784	1.88004	-4.33779	3.12210
	Equal variances not assumed			-.323	98.787	.747	-.60784	1.88004	-4.33835	3.12266
